# Pre-service mathematics teachers’ integration of design thinking in STEM lesson plans: Insights from a qualitative case study

**DOI:** 10.1371/journal.pone.0361875

**Published:** 2026-11-02

**Authors:** Rüveyda Karaman Dündar

**Affiliations:** Department of Mathematics and Science Education, Bartin University, Bartin, Türkiye; Tel Aviv University, ISRAEL

## Abstract

Mathematics is foundational in science, technology, engineering, and mathematics (STEM) education. Mathematics can foster problem-solving, quantitative reasoning, modeling, and creativity across disciplines. This study explores how pre-service mathematics teachers (PMTs) in a Design Thinking (DT)-focused course integrate DT into STEM lesson planning, the factors influencing this integration, and the challenges encountered during lesson planning. 55 senior PMTs at a state university in Türkiye participated in the study. Data were collected through DT-oriented, mathematics-focused STEM lesson plans and an open-ended questionnaire and were analyzed using content analysis. Findings reveal that while many PMTs maintained mathematics as a core component in their plans, the integration of DT was often partial and unbalanced. The Define, Ideate, and Prototype phases were predominant across most plans (>80%), whereas the Empathy phase was considerably less represented, appearing in only 20.8% of plans. In contrast, while the Test phase was incorporated into more than half of the lesson plans (54.2%), its presence remained substantially lower than that of the initial construction phases, reflecting a tendency toward linear product completion rather than sustained, iterative design optimization. Participants reported difficulties aligning DT phases with learning objectives, selecting appropriate materials, and designing effective assessment strategies. Despite these challenges, PMTs strongly believed in DT’s potential to enhance student engagement, creativity, and real-world mathematical thinking. The study underscores the need for teacher education programs to provide more explicit scaffolding and practical tools to meaningfully embed all phases of DT within mathematics instruction.

## Introduction

The rapid technological advances of the 21st century have intensified global efforts to strengthen STEM education, with increasing emphasis on problem-solving, analytical reasoning, creativity, and collaboration [1,2]. Consequently, many countries have implemented STEM reforms that promote interdisciplinary learning to prepare students to address complex, real-world challenges [3–6]. Despite these efforts, research suggests that STEM disciplines are not always integrated equitably, with mathematics often receiving limited attention in interdisciplinary activities [7–9]. This is concerning because mathematics provides essential tools for modeling, analysis, and logical reasoning across STEM disciplines [10–12]. Moreover, when connections between mathematics and other STEM fields are not made explicit, students may have difficulty recognizing the relevance and value of mathematical learning [6]. Thus, the purposeful integration of mathematical reasoning is essential for fostering coherent and meaningful interdisciplinary STEM learning [12].

One promising approach to strengthening integration, particularly between mathematics and other STEM disciplines, is Design Thinking (DT). DT is a human-centered, iterative framework for addressing real-world problems through empathy, problem definition, ideation, prototyping, and testing phases [13,14]. As a pedagogical approach, DT promotes inquiry-based learning, creativity, systems thinking, collaboration, intellectual risk-taking, and reflection [2,15]. Although rooted in engineering, DT is increasingly recognized as a cross-disciplinary approach applicable to science, mathematics, and technology [6,16,17]. In mathematics education, DT can foster meaningful learning by positioning students as designers who engage in mathematical modeling, abstract reasoning, and iterative refinement [18,19]. Through contextualized, design-oriented tasks, students can explore mathematical concepts while applying them to complex, authentic problems.

Despite this potential, DT remains underutilized and under-researched in mathematics education, particularly at the primary and middle school levels [11,20,21]. Although young learners can successfully engage in DT with appropriate guidance [22,23], pre-service mathematics teachers (PMTs) often receive limited preparation for incorporating DT into instructional planning [24]. Moreover, teacher education programs frequently lack instructional models that promote student agency, idea generation, collaboration, and design-oriented learning [8,16,21]. Given DT’s potential to strengthen mathematical reasoning while fostering creativity, collaboration, and resilience [25], addressing this gap in teacher preparation is important for promoting the meaningful integration of DT in mathematics and STEM education.

This study addresses these empirical gaps by examining how PMTs, in a course explicitly focused on design thinking, conceptualize and implement DT in STEM lesson planning. Specifically, it explores their pedagogical strategies, the challenges they encounter, and their views on integrating mathematics and DT in instructional contexts. Through this investigation, the study contributes to the broader discourse on preparing teachers to support interdisciplinary, DT-oriented STEM learning that elevates mathematics’ role.

The research questions are:

1. How do pre-service mathematics teachers, in a course explicitly focused on design thinking, integrate mathematics and phases of design thinking into STEM lesson plans?
2. What factors influence pre-service mathematics teachers’ selection of design thinking-oriented topics, and what challenges do they face in developing these lesson plans?
3. What are pre-service mathematics teachers’ views on integrating design thinking and mathematics in STEM education?

The problem space addressed by this study is the philosophical vagueness and disciplinary inequity inherent in integrated STEM, specifically the tendency for mathematics to be overshadowed by science-dominated models [2,7]. While integrated STEM is widely advocated, scholarly debate persists regarding how student learning outcomes arise from specific disciplines within integration [7]. This study addresses the unresolved issue of how future educators navigate the tension between maintaining mathematical rigor and employing iterative design processes. It seeks to determine whether DT can serve as an effective operational mechanism for making mathematics transparent and explicit.

## Theoretical framework

### Integrated STEM education

Integrated STEM education emphasizes meaningful connections among science, technology, engineering, and mathematics through authentic, problem-based learning. Moore et al. [26] define integrated STEM as the combination of two or more STEM domains to support deep, contextualized learning. Rather than merely bringing multiple disciplines together, integrated STEM involves epistemic connections among disciplinary knowledge and practices through authentic problem contexts, design inquiry, and iterative learning processes [7,26]. This perspective aligns with situated cognition theory [17,27,28], which emphasizes learning in authentic, socially mediated contexts where knowledge is shaped through participation and interaction. By engaging learners in authentic problem-solving activities that require interdisciplinary knowledge, social interaction, and iterative inquiry, integrated STEM education operationalizes key principles of situated cognition and fosters contextualized, meaningful learning.

Hallström and Ankiewicz [2] identify two modes of interaction within integrated STEM: applying existing knowledge from individual disciplines and concurrently developing new expertise across domains. Design, particularly DT, emerges as a shared methodology that facilitates both modes. As a pedagogical strategy, engineering design promotes cross-disciplinary learning by integrating scientific inquiry and mathematical reasoning [6,29]. DT fosters deep understanding and real-world application of STEM concepts [2,11,20], ultimately supporting the development of critical interdisciplinary competencies required for future STEM careers [6]. In this study, design is conceptualized as a broad pedagogical approach in which interdisciplinary STEM learning occurs through extended design tasks and the creation of artifacts. In contrast, design thinking is treated as a specific design framework that structures these tasks through iterative, user-centered phases, including empathy, ideation, and testing. This distinction allows for a clearer examination of how PMTs organize design processes and integrate mathematics within design-oriented STEM instruction.

Drawing primarily on the principles of integrated STEM [7], the framework in the current study conceptualizes meaningful STEM integration through interconnected analytical dimensions that make the notion of integration explicit and observable in lesson plans. In the first dimension, integration is conceptualized along a continuum, allowing lesson plans to be examined in terms of their depth of integration rather than treated dichotomously as integrated or not. At the *multidisciplinary* level, disciplines remain largely separate yet connected by a shared theme or context. At the *interdisciplinary* level, concepts and practices from two or more disciplines are purposefully coordinated to deepen understanding. At the *transdisciplinary* level, disciplinary distinctions become secondary to addressing authentic real-world problems, with knowledge and practices from STEM disciplines jointly shaping the learning experience. The second dimension addresses disciplinary equilibrium, responding to critiques that integrated STEM often defaults to science-dominated models.

### The role of mathematics in STEM education

Although mathematics is a cornerstone of STEM [30], it is often underrepresented, especially in elementary and integrated STEM settings, where science tends to dominate instructional practices [7,8,31–34]. Strengthening the mathematics component requires positioning mathematical reasoning at the core of STEM activities to foster problem-solving, critical thinking, and adaptive reasoning [33,35,36]. English [11] emphasizes that mathematics serves multiple functions within STEM: as a foundational disciplinary core, a mode of critical reasoning, and a primary tool for modeling that structures interdisciplinary integration. Mathematical thinking supports the evaluation of claims, the analysis of arguments, and evidence-based justification, while mathematical modeling enables learners to develop STEM concepts through authentic, complex problem-solving [7,11,37–40]. Accordingly, strengthening mathematics in STEM through mathematical modeling can promote creativity, analytical thinking, 21st-century competencies, and responsible citizenship in response to contemporary challenges [8,29,37].

To meet the demands of an increasingly complex world, mathematics education must shift toward interdisciplinary, design-oriented instruction that nurtures deep, transferable skills [11,41]. Engineering DT provides a compelling framework for such integration, enabling students to engage in mathematical modeling while solving real-world problems [2,11]. However, without deliberate efforts to highlight the relevance of mathematics within interdisciplinary STEM activities, students may overlook its value in practical problem-solving [6,12]. Emerging research [16] supports the view that integrated STEM, especially grounded in DT, can enrich mathematics education by offering engaging and authentic learning experiences [30,42,43].

To provide analytical focus, this study distinguishes between four interrelated constructs. First, mathematics as a discipline refers to the foundational core of content knowledge [11]. Second, mathematics as a component of STEM is conceptualized as an epistemic partner that plays a reciprocal role by informing decisions and constraining designs within a problem space [7,11]. Third, mathematical thinking denotes the specific cognitive processes of reasoning, abstraction, and evidence-based justification required to navigate complexity. Finally, mathematical modeling is defined as the process of integration. It is the process of developing conceptual innovations in response to real-world needs, serving as the bridge between abstract theory and physical application. Rather than advancing a purely normative claim that mathematics must be at the core of STEM, this study adopts a compensatory analytical stance necessitated by current research trends. Because mathematics is frequently overshadowed in integrated STEM settings [7,11,44], this marginalization may hinder students’ development and application of quantitative reasoning for efficient problem solving. Therefore, our focus on mathematical centrality is a deliberate research design choice intended to test whether DT can serve as a successful operational mechanism to make the discipline transparent and explicit [7].

### Design thinking in integrated STEM education

DT is critical for supporting interdisciplinary STEM education, particularly by embedding mathematical reasoning within iterative, real-world problem-solving contexts. DT, as described in the Stanford d.school framework [45,46], consists of five iterative phases: Empathy (or Empathize), Define, Ideate, Prototype, and Test (Fig 1). These stages emphasize user-centered problem-solving and creativity, making DT an essential component of integrated STEM education and central to engineering practices [21,47].

**Fig 1:**
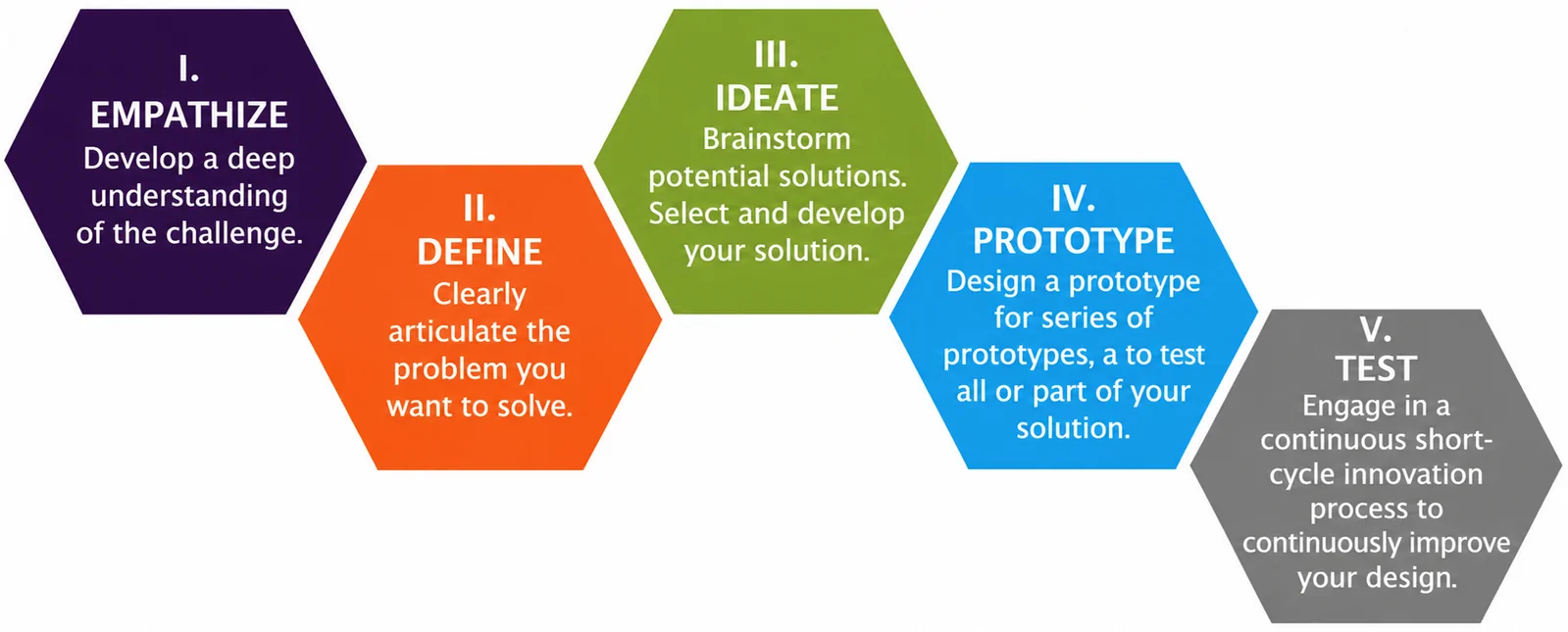
Stanford d. school DT method [33].

The design thinking process should not be conceptualized as a strict, linear sequence; rather, it operates as a flexible and non-linear framework. In practice, students may move flexibly and nonlinearly between design thinking phases in response to the evolving demands of a project. For example, feedback from testing may reveal new insights, prompting students to loop back to the definition phase to readjust their problem statement before returning to prototype development. Ultimately, successful engagement with design thinking requires continuous iteration across multiple stages [45].

DT has been adopted as a key mechanism for STEM integration in various international contexts. For instance, Singapore's STEM-Applied Learning Programme incorporates scientific inquiry and DT to promote student engagement [11,48]. Likewise, the U.S. Next Generation Science Standards prioritize engineering design practices for all learners [49,50]. The process of learning while designing enhances STEM learning by blending theoretical understanding with practical and iterative experiences [2,18]. DT fosters perseverance and resilience, allowing students to apply scientific and mathematical concepts in meaningful contexts [11,51,52].

Although mathematics is sometimes perceived as rigid or rule-bound, mathematical thinking can support design processes and creative problem-solving. Through DT-based activities, students engage in sense-making and develop analytical thinking in mathematics and broader STEM contexts [11,19,33]. Traditional barriers between mathematics and engineering can be overcome by reimagining mathematics as a creative, design-driven process [20,53]. DT's iterative nature promotes flexible problem-solving strategies, including ideation, prototyping, and refining, which cultivate a growth mindset [14,15,54]. DT also enhances collaboration, confidence, and engagement when integrated with mathematical modeling, game-based learning, and design challenges [25,54–56]. Moreover, it supports diverse learners, particularly those who benefit from hands-on, exploratory environments [57]; however, challenges related to curriculum alignment and supporting students’ confidence must be addressed [14].

Drawing on English [11], mathematical thinking, specifically through reasoning, abstraction, and modeling, is expressed across the iterative phases of design thinking as follows:

1. Empathy: The first phase is user-centered, focusing on understanding the needs and perspectives of the intended users. At this stage, feedback is gathered, and the situation is explored to develop a deep understanding of users’ experiences. In this initial phase, referred to in [11] as problem scoping and problem interpretation, mathematical thinking is used to evaluate the constraints and requirements of a real-world situation.
2. Define: This phase involves identifying and scoping the problem, articulating the core problem, and evaluating the specific constraints and requirements of a real-world situation based on information gathered during the Empathy phase. In this phase, students use reasoning and critical mathematical thinking to evaluate statements and claims related to the problem.
3. Ideate: This phase marks the transition from abstract concepts to potential design solutions and encourages students to develop innovative ideas beyond standard textbook approaches. During idea generation, mathematical thinking supports this process through abstraction and modeling, as students draw on mathematical knowledge, including geometric properties and spatial reasoning, to formulate and explore initial design ideas.
4. Prototype: During this phase, students translate their ideas into physical or conceptual models that make their thinking visible. As English [11] suggests, creating a design can serve to represent and externalize students’ thinking. In mathematics education, constructing a prototype can provide opportunities for students to apply and demonstrate their mathematical reasoning and conceptual understanding. For example, designing an optical instrument may require students to apply their knowledge of geometric angles to develop and refine a functional design.
5. Test: This phase involves evaluating a prototype against predefined criteria and constraints through systematic testing, reflection, and iterative refinement. Mathematical thinking supports this process through measurement, quantitative analysis, and evidence-based reasoning. Students collect and analyze performance data to determine whether their designs meet the specified requirements and to identify areas for improvement. For example, when designing an aircraft model, students may use mathematical modeling to investigate how changes in wingspan affect other design parameters, such as fuselage dimensions. Based on their findings, students revise their models and evaluate the consequences of these modifications. This iterative process encourages adaptive reasoning and informed decision-making, enabling students to refine their designs and develop a deeper understanding of the mathematical relationships underlying their solutions.

### The role of pre-service mathematics teachers in integrated STEM education

PMTs’ preparation and beliefs shape how they integrate interdisciplinary approaches and mathematical reasoning into their teaching practices. Research suggests that engaging pre-service teachers in STEM experiences can foster their appreciation of meaningful mathematics learning, mathematical discourse, and sense-making [16,58]. However, integrating mathematics into interdisciplinary STEM contexts can be challenging when mathematics is perceived as static knowledge rather than a dynamic, inquiry-oriented discipline [53]. Immersive teacher preparation programs that engage pre-service teachers in authentic, real-world STEM contexts may help bridge the gap between theoretical knowledge and classroom practice [58–61]. In this context, DT, with its emphasis on iterative design, problem solving, and idea refinement, offers a potentially valuable approach to teacher preparation [60].

### Research gap

While DT offers opportunities to integrate mathematics into STEM education by fostering creativity, reasoning, and problem-solving, its use remains limited in practice, particularly in K–12 education [11,19,61–63]. DT's potential to promote deep, interdisciplinary learning is well documented [20], and evidence suggests that even young children can engage meaningfully in DT when appropriately supported [22,23]. However, mathematics remains underrepresented in many STEM contexts, especially in early education [11]. Addressing this challenge requires further research into how DT can be incorporated into school curricula and how pre-service teachers can be prepared to design and implement interdisciplinary learning experiences that meaningfully integrate mathematics with other STEM disciplines [19,20,25].

PMTs are pivotal in this effort, as their ability to connect DT with mathematical sense-making directly impacts student learning [16]. However, pre-service teachers often struggle to integrate STEM concepts because of limited interdisciplinary exposure and rigid disciplinary frameworks [24,47,61]. Although DT is gaining recognition, effective pedagogical strategies for integrating it into mathematics instruction remain underexplored [56], particularly regarding how DT activities develop teachers’ DT skills [52] and how mathematics can function as a driver of innovation in interdisciplinary settings [62]. While research on DT mindsets and general instructional practices in STEM is growing [16,64], the conceptual coherence required to position mathematics centrally within DT remains insufficiently examined.

The ways of thinking that teachers use in STEM-focused learning environments remain underexplored, with a notable lack of theoretical frameworks to guide this understanding. Although DT is often presented as a framework for connecting disciplines, how it is incorporated into primary and middle school mathematics education and how mathematical reasoning supports its implementation need further investigation [11].

Some existing studies emphasize structured interventions, leaving room for further exploration of DT's open-ended, iterative nature and the challenges teachers may encounter in implementing it [64].

Although previous research has examined pre-service teachers’ general STEM competencies [24,61,65], less attention has been paid to how they translate mathematical knowledge and reasoning into DT-based STEM lesson plans. In particular, further research is needed to understand how pre-service mathematics teachers incorporate mathematical reasoning into design activities and how they address the iterative processes central to DT [2,7,64]. Investigating these issues can provide insight into the opportunities and challenges associated with preparing teachers to design interdisciplinary STEM learning experiences in which mathematics serves an integral role rather than merely providing contextual support.

To address these gaps, this study examines how pre-service mathematics teachers integrate DT into mathematics-focused STEM lesson planning, with particular attention to their approaches to mathematical reasoning, the factors influencing their planning decisions, and the challenges they encounter. Using an interpretive case-study approach, the study explores how participants conceptualize the relationship between mathematics and other STEM disciplines and how this understanding is reflected in their lesson plans and reported experiences. The findings contribute to research on interdisciplinary STEM teacher preparation by providing insight into how pre-service mathematics teachers incorporate DT into lesson planning and the extent to which their plans reflect meaningful mathematical integration and iterative design processes.

## Materials and methods

### Participants

Participants were 55 senior pre-service mathematics teachers (33 female, 22 male) enrolled in an elementary mathematics education program at a state university in Türkiye. Purposive sampling was used to select participants who met the following criteria: being in their senior year, attending course sessions regularly, voluntarily agreeing to participate in the study, and actively engaging in course activities, including assignments and lesson plan development.

Selecting senior PMTs was appropriate for the study because they had completed substantial coursework in mathematics and mathematics education and were approaching entry into the teaching profession. Their preparation provided a relevant context for examining how prospective teachers integrate mathematical reasoning and design thinking into STEM lesson planning. Regular attendance and active participation in course activities were considered important for ensuring that participants had sufficient exposure to the 15-week instructional sequence and opportunities to engage with its content.

This study was conducted in accordance with ethical standards for research involving human participants. Ethical approval was obtained from the Bartin University Social and Human Sciences Ethics Committee (Protocol No: 2021-SBB-0302). All participants provided informed and written consent before data collection. None of the participants had prior experience with DT or STEM-focused coursework. To mitigate potential power imbalances inherent in the instructor-researcher role, recruitment emphasized the voluntary and non-evaluative nature of the research. Participants were informed that their decision to participate or to withdraw at any time would have no impact on their course grades or their relationship with the instructor.

### Research context

This study employed a **qualitative case study design** to explore PMTs’ views, challenges, and instructional approaches to developing DT-oriented STEM lesson plans. A case study is appropriate for investigating a particular group, program, or process within its real-life context [66,67]. Accordingly, the case was bounded by a single cohort of PMTs participating in a methods course focused on integrating DT into mathematics education through STEM lesson planning. This design enabled an in-depth examination of participants’ experiences and reflections within the specific instructional context.

This study employed a single-case embedded design [67], drawing on the descriptive and heuristic traditions of case study research to examine participants’ experiences and meaning-making within a specific instructional context [66,68]. The case study design allowed for a within-case synthesis that captured both collective instructional outcomes (group lesson plans) and individual pedagogical sense-making (reflective questionnaires) [67]. This design enabled an examination of how PMTs incorporated DT into mathematics-centered STEM lesson planning and the challenges they encountered [69]. The purposive sample supported the investigation of the DT stages in mathematics-centered design by providing a cohort that was theoretically prepared for integration but procedurally novice in DT. By selecting engaged seniors, the study could focus on conceptual and pedagogical barriers to integration rather than on gaps in basic mathematical knowledge or lack of exposure to the course materials.

The author designed a 15-week course with weekly 2-hour sessions to develop pre-service teachers’ theoretical understanding and practical skills in DT and STEM education, emphasizing a mathematics-centered approach (Table 1). The case is bounded spatially and temporally by the methods course conducted during the 2021–2022 academic year. The primary unit of analysis is the instructional experience of the senior PMTs. Within this single case, the study employs embedded units of analysis to capture the multi-layered nature of teacher planning: (1) The design group serves as the unit of analysis for the lesson plan artifacts (n = 24), reflecting collective instructional decision-making, and (2) The individual PMT serves as the unit of analysis for the reflective questionnaires, capturing personal interpretations and perceived challenges.

**Table 1:** Weekly instructional sequence and core activities of the 15-week methods course.

Week 1	Course objectives, structure, and assessment explained.
Week 2–4	Discuss associative reasoning in mathematics, exploring relational/operational understanding and concept hierarchies.
Week 5–6	Introduction to interdisciplinary connections and STEM theory
Week 7–8	Introduction to DT framework; mid-term exam
Week 9–10	Analysis of sample DT-STEM tasks; group formation; lesson planning task assigned
Week 11–14	In-class group presentations of lesson plans with peer and instructor feedback
Week 15	Submission of final lesson plans; self-evaluation questionnaire administered

The researcher guided the participants in developing their capacity for designing DT-oriented STEM lessons with a mathematical focus. After the fifth week, the researcher facilitated sessions in which the PMTs explored the history and theoretical foundations of STEM education through assigned readings and class discussions. Subsequently, the PMTs were introduced to the principles and stages of DT and examined sample activities adapted from [45] and research publications such as [33]. These examples served as models for integrating DT into mathematics-focused STEM instruction. Working collaboratively in groups of two or three, the PMTs designed their own DT-oriented STEM lesson plans that emphasized mathematical concepts. Each group presented its lesson plan to the class, engaged in peer and instructor feedback sessions, and revised its plan accordingly. At the conclusion of the course, the instructor evaluated the final lesson plans, and each PMT completed a self-evaluation questionnaire reflecting on their design process and learning outcomes.

A total of 24 lesson plans were developed to integrate mathematics with other STEM disciplines. For the lesson plan development, participants were required to use a standardized lesson plan template designed by the researcher. This template explicitly included sections corresponding to the five phases of design thinking. The directions instructed PMTs to organize and label their lesson plans according to these phases and to describe how each phase would be implemented within the instructional sequence. This structure ensured that the DT framework guided their lesson design process and provided a consistent basis for analyzing how each phase was addressed across lesson plans.

Topics in the lesson plans included, but were not limited to, constructing a solar system model, designing a periscope, and solving real-world optimization problems through mathematical modeling (see Table 2 for details).

**Table 2:** Overview of PMT-developed STEM lesson plans: Design topics, mathematical objectives, and target grade levels (N = 24).

Lesson Plan #	Title of the Lesson Plan	Math Objectives in the Lesson Plan	Grade Level
1	Designing a magnifying glass	Reflecting on experiences gained regarding the properties of polygons	5th
2	Kite design	Designing different geometric shapes, length measurements, and area formulas.	7th
3	Designing an irrigation system to manage rainwater for the garden plants optimally	Analogical reasoning about the volume relationship of a right circular cylinder is based on deriving the area relationship of a circle.	8th
4	Model well construction	Being able to reason about ratio relationships based on real-life situations	7th
5	Environmental awareness and recycling	Applying experiences with the surface development of a right circular cylinder.	7th
6	Kite design	Being able to work with categorical data and make data-driven decisions.	5th
7	Freezer design	Working with categorical or discrete (quantitative) data and making data-driven decisions.	5th
8	Square pattern design	Being able to work with categorical data and make data-driven decisions.	5th
9	Landslide: Trees and slope	Interpreting proportional situations based on real-life scenarios.	7th
10	Designing a thermometer	Interpreting natural numbers, integers, and rational numbers in real-life or mathematical situations.	7th
11	Designing a wind turbine	Interpreting proportional situations based on real-life scenarios.	7th
12	Making disinfectant with natural materials	Reasoning about ratio relationships through real-life situations.	7th
13	Designing a colored ball	Being able to determine and make decisions based on the most appropriate probability approach (experimental) for an event encountered in real-life	8th
14	Designing solar system (World, Moon, and Sun Model)	Geometric concepts related to scale, distance, and angles. Scale and distance calculations.	8th
15	Designing a three-dimensional illusion display based on types of visual illusions	Drawing two-dimensional views of three-dimensional objects from different perspectives.	7th
16	Constructing an earthquake-resistant, high-rise building	Formulates the volume formula for a rectangular prism and solves related problems.	6th
17	Creating objects from geometric solids	Recognizes right prisms, identifies their basic elements, constructs them, and draws their surface developments.	8th
18	Roof design	Recognizes the right pyramid, identifies its basic elements, constructs it, and draws its development.	8th
19	Ship design	Ability to work with categorical or quantitative (discrete) data and make data-driven decisions	6th
20	Creative drama	Studies involving real-life situations related to comparing and ordering integers	6th
21	Designing greenhouses from different materials	Working with categorical data and making data-driven decisions.	5th
22	Designing costumes using recycled materials with the help of geometric shapes	Analyzing the surface developments of right prisms, right rectangular pyramids, right circular cylinders, and right circular cones.	8th
23	Watch design	Making data-driven decisions	5th
24	Periscope design	Applying geometric concepts related to angles, reflections, and measurements.	7th

Table 2 demonstrates that the PMTs integrated engaging topics into their lesson plans, many extending beyond mathematics to encompass other STEM disciplines. The lesson plans targeted a range of middle school grades and addressed multiple mathematical domains, including numbers and operations, algebraic thinking, geometry, the statistical research process, and data and probability.

For example, one group developed a lesson, Designing *an Irrigation System*, targeting 8th-grade students. The mathematical focus was on exploring the volume of a cylinder using analogical reasoning from the area of a circle. Students were required to calculate optimal container shapes to efficiently collect rainwater, a task that integrated environmental science, measurement, and engineering design within a DT framework.

### Data collection

Data were collected from multiple sources to obtain an in-depth, triangulated understanding of the PMTs’ engagement in DT-oriented STEM lesson design. The first data source was the DT-oriented STEM lesson plans collaboratively developed by the PMTs, which were collected to provide direct evidence of how DT phases and mathematical content were operationalized in instructional designs. The second data source was a semi-structured, open-ended questionnaire designed to elicit participants’ reflections on their experiences, pedagogical reasoning, and the perceived challenges and affordances of DT integration throughout the lesson-planning process.

In crafting the questionnaire, a thorough review of the relevant literature was conducted to ensure that the questions aligned with the study's objectives and could capture essential data. This literature review guided the formulation of pertinent questions and ensured that they addressed significant constructs identified in existing research. The questionnaire was informed by relevant literature on interdisciplinary planning, DT, and pre-service teacher education. Each item was explicitly mapped to constructs emphasized in prior research, such as content selection and curricular alignment [45,57], challenges and affordances of DT implementation [14,15], anticipated learning outcomes [54], and teachers’ pedagogical competence in DT-oriented instruction [24], thereby ensuring conceptual alignment between the instrument and existing literature. The questionnaire was not based on a pre-existing instrument but was instead developed contextually to align with the course objectives. Its content validity was established through consultation with three experts in mathematics education and STEM pedagogy, each with doctoral-level expertise and publications in STEM curriculum development and teacher training. Based on their feedback, several prompts were refined for clarity and depth.

The questionnaire was pilot tested with a separate group of PMTs (n = 8), and modifications were made based on clarity, relevance, and response richness. In line with qualitative paradigms, the trustworthiness of the data was emphasized through expert validation, prolonged engagement, peer debriefing, and participant reflection. The prompts were refined through an iterative validation process to enhance their clarity and elicit richer, multidimensional responses. For example, in the initial draft, Q1 was phrased as, “What is your lesson topic?” Following a pilot administration with eight PMTs, it was expanded to “How did you determine the content for your lesson plan, and why did you choose it?” Similarly, Q5 was initially phrased as, “Do you think Design Thinking is useful for math?” Following expert review, it was revised to, “What are the benefits of designing DT-oriented STEM lessons?” This revision was intended to encourage participants to reflect on the potential benefits for students, teachers, and disciplinary learning rather than provide a binary response. The pilot indicated that participants benefited from a more specific prompt that encouraged them to articulate connections between their personal experiences and the mathematical objectives of their lesson plans.

The final questionnaire consisted of eight open-ended prompts designed to probe PMTs’ lesson design rationale, experiences with DT integration, anticipated student learning outcomes, and views on curriculum alignment:

- How did you determine the content for your lesson plan, and why did you choose it? (This question is about topic selection and grounded in the concept of STEM volition [2,45,57], probing the intent behind the chosen context.)
- What challenges did you encounter in planning a DT-oriented STEM lesson? (This question is about planning challenges and informed by Kim and Bolger’s findings on the barriers to interdisciplinary integration [61].)
- What student outcomes do you anticipate from the lesson you designed? (This question is about anticipated outcomes and is mapped to English’s ways of thinking framework [11], specifically identifying if PMTs envisioned students as routine or adaptive problem solvers [54].)
- How do you envision learning occurring through your lesson plan? (This question concerns learning processes and is mapped to English’s ways of thinking framework [11], specifically identifying whether PMTs envisioned students as routine or adaptive problem solvers [54].)
- What are the benefits of designing DT-oriented STEM lessons? (This question concerns pedagogical benefits and is based on research regarding the affordances of DT for student engagement [2,6,7,11,20].)
- Do you believe mathematics teachers should be skilled in DT-based lesson planning? (This question concerns teacher skills and investigates the pedagogical competence required to maintain mathematical rigor within DT [8,16,21–25].)
- Does DT align with the needs of the national mathematics curriculum? (This question concerns curricular alignment and addresses the tension between the cyclical nature of design and the linear nature of school curricula [8,16,21–25,45,57].)
- What are the strengths and limitations of using DT in STEM education? (This question concerns strengths and limitations and elicits PMTs’ critical evaluation of DT as a methodological backbone for STEM [2,7,14,15].)

All participants completed the questionnaire via Microsoft Forms or hard copies in the course's final week. The participants were informed of the study's aim and the significance of their responses. Participants were given one week to submit their responses. Although lesson plans were produced collaboratively in groups, questionnaire responses were collected **individually**, allowing for the examination of variation in personal interpretations and experiences within shared design processes. To enhance trustworthiness, multiple strategies were employed, including expert validation, pilot testing, prolonged engagement with the data, peer debriefing, and reflexive analytic memoing throughout the analysis process.

### Data analysis

Data analysis proceeded in two complementary strands corresponding to the two data sources: systematic analysis of DT-oriented STEM lesson plans and qualitative content analysis of questionnaire responses.

#### Analysis of DT-oriented STEM lesson plans.

The collaboratively developed lesson plans (n = 24) were analyzed to examine the extent to which STEM integration and mathematical emphasis were realized in DT-oriented designs. To evaluate the STEM features of the lesson plans developed by the participants, the rubric established by Kim and Bolger [61] was employed. This rubric assesses six core STEM features: applicability to student level, subject interdependence, originality of application, math-focused integration, essential STEM inclusion, and differentiation between technology and engineering. This evaluative tool was instrumental in addressing the research question about PMTs’ integration of mathematics into STEM lesson plans. Each lesson plan was independently evaluated by two raters, the primary researcher and a mathematics education expert. Each STEM feature in each lesson plan was evaluated using a binary scoring system (1 = robust lesson plan; 0 = simple or insufficient). Table 3 presents the assessment criteria used in the evaluation. Prior to the discussion, the initial independent inter-rater agreement was 93.1%. Discrepancies were resolved through evidence-based discussion and refinement of criterion boundaries, resulting in consensus for the final dataset, consistent with established qualitative reliability procedures [70].

**Table 3:** Evaluative criteria and operational rubric for STEM integration features in lesson plans (adapted from Kim & Bolger [61]).

STEM Features	Robust Lesson Plan	Simple Lesson Plan
Applicable level for students	The S, T, and E topics are applicable to students’ level (Using the textbook for the relevant grade level).	Some of the S, T, and E content is not appropriate for students’ level (Usually at an inadequate level).
Subject interdependence	The S, T, and E content facilitates a better understanding of the M topic.	The inclusion of S, T, and E content does not change how M will be comprehended.
Original application	Students are encouraged to draw innovative or original connections to the real world by including science, technology, and engineering in their studies.	The only purpose of S, T, and E is to aid the student in understanding the concept of textbook mathematics.
Math-focused	T, E, and S are integrated into math-related tasks, with M being the focus.	The lesson primarily emphasizes S, T, or E rather than mathematics.
Essential STE inclusion	The mathematics topic effectively incorporates more than two S, T, and E concepts.	The mathematics lesson only uses one S, T, or E concept.
Differentiating T from E	The goal of the lesson is to help students comprehend E and T.	Evidence of the mixing of the T and E components.

The first feature ensures that students’ grade levels are taken into account when selecting content across subjects. For instance, a lesson plan created for a specific grade might include mathematics content appropriate for that level while addressing other subjects at a lower level. The second feature involves integrating interrelated STEM topics, thereby enhancing cross-subject understanding. The third feature emphasizes promoting students’ creative application to real-world problems. This approach fosters their ability to explore authentic situations from multiple perspectives and encourages a sense of inquiry. The fourth feature focuses specifically on mathematics, while the fifth requires including at least two additional disciplines alongside mathematics in the lesson plan. The final feature distinguishes between T and E: while T represents the stable elements of concepts or products, E involves using mathematics to create highly optimized products that benefit humanity [61].

During data analysis, each lesson plan was also coded according to the presence and quality of the five DT phases outlined in the template. The Stanford d.school [46] design thinking model serves as the analytical framework for examining how DT phases and iterative processes are represented in pre-service mathematics teachers’ lesson plans. Situated cognition theory complements this framework by conceptualizing learning as context-dependent activity in which disciplinary knowledge and tools are used to address authentic problems. From this perspective, meaningful STEM integration requires the purposeful coordination of disciplines within a shared problem-solving context. Mathematics contributes through modeling, logical reasoning, and quantitative analysis that inform design decisions, while DT provides opportunities to apply and refine mathematical knowledge through iterative design. Together, these frameworks support an analysis of how PMTs integrate DT into STEM lesson planning and the extent to which their plans connect mathematical reasoning with authentic problem-solving.

To ensure methodological transparency and address the distinction between structural occurrence and disciplinary quality, the coding protocol differentiated between nominal mention and substantive implementation. A score of 1 (robust implementation) was assigned when a phase was not only explicitly planned in the instructional sequence, but also operationalized mathematical reasoning as an analytical and integrative driver (e.g., using mathematical data modeling to evaluate prototype constraints during testing, or applying geometric properties to define the design boundary). A score of 0 (absent or superficial) was assigned under two distinct conditions. First, structural absence, where the phase was entirely omitted from the lesson sequence and template, and second, superficial presence, where the phase was labeled or described as a generic arts-and-crafts activity without incorporating mathematical reasoning, quantitative analysis, or evidence-based justification. The distinction between robust and simple enactment was grounded in the study’s theoretical framework. Specifically, the designation of a phase as robust was informed by prior conceptualizations in the literature (e.g.[11,33],), which emphasize mathematics as a unifying structure within design thinking processes. Accordingly, robustness was operationalized as the meaningful use of mathematical reasoning as an analytical and integrative tool rather than as a superficial reference.

Operational definitions guided DT phase coding, and exemplar-coded lesson plan excerpts are presented in the Results section to illustrate coding decisions and support analytic reliability. To preserve the integrity of both dimensions, the raters maintained an audit trail distinguishing purely omitted phases from those that were present but lacked mathematical rigor. This operational definition ensures that phase frequencies not only capture formal template compliance but also reflect genuine, mathematics-centered pedagogical enactment.

The analysis further demonstrated that higher design thinking scores were consistently associated with instances in which mathematics was actively employed across multiple phases of the design process, particularly during testing and refinement. This pattern provided empirical support for the interpretive distinction between simple phase occurrence and substantive mathematical integration. Therefore, phases were not coded as present merely on the basis of reference; rather, their inclusion in the scoring system required evidence of meaningful mathematical engagement to be considered robust.

The use of two complementary frameworks allowed us to capture the multidimensional nature of PMTs’ STEM lesson plans. Kim and Bolger’s [61] rubric was employed as an analytical lens to assess the quality of STEM integration, with criteria such as subject interdependence and creative application used to determine whether the lesson plans reflected meaningful and substantive integration across STEM disciplines. This was complemented by Bush et al.’s [33] framework, which provided the procedural structure of the design process through its five-phase cycle. The framework was used to analyze how PMTs progressed through the design process, with mathematics positioned as the analytical driver of the tasks. Together, the frameworks enabled the evaluation of both interdisciplinary integration and disciplinary rigor in the resulting lesson plans.

#### Analysis of questionnaire data.

The qualitative analysis of the questionnaire data was conducted using a bottom-up thematic approach to ensure that the findings accurately represent the phenomena under study and provide the reader with confidence in the resulting interpretations. As Yin [67] emphasizes, a robust research design serves as a logical model of proof, where the goal of reliability is to minimize errors and biases in a study. By following explicit procedures, we ensured that the study's operations, specifically the translation of raw data into analytical themes, could be repeated by a later investigator with identical results.

The qualitative questionnaire data were analyzed inductively using an iterative four-stage thematic approach: familiarization, initial coding, category development, and theme construction. Coding was primarily conducted by the first author, with a second qualitative researcher specializing in mathematics education participating in reliability and validation procedures to minimize individual interpretive bias and establish an auditable chain of evidence [52,53,67–69,71].

First, the researchers independently conducted multiple readings of the raw questionnaire responses to develop a comprehensive understanding of PMTs’ experiences and perspectives [72]. Second, recurring words, phrases, and meaning units were identified and assigned descriptive initial codes that remained closely aligned with participants’ original language to maintain interpretive transparency and minimize premature abstraction. Third, conceptually related codes were systematically clustered into broader categories, including the rationale for topic selection, logistical constraints, and pedagogical affordances. Finally, these categories were synthesized into overarching themes aligned with the study’s research questions, thereby providing a detailed account of the instructional reasoning underlying the PMTs’ responses [68,72]. To enhance analytic transparency, the progression from raw data to themes was systematically documented. Table 4 presents exemplar quotations alongside initial codes, categories, and final themes, illustrating how the themes were derived from participants’ responses.

**Table 4:** Progression from raw participant quotations to initial codes, categories, and final themes.

Exemplar Participant Quotation (Raw Data)	Initial Code	Category	Theme
“We had difficulty deciding how to integrate science and math meaningfully, especially with limited knowledge about engineering.”	Difficulty with interdisciplinary integration	Planning challenges	Challenges to incorporating DT in STEM lesson plans
“The most challenging part was aligning the design with other disciplines and finding suitable learning outcomes”	Difficulty aligning disciplines and outcomes	Interdisciplinary planning challenges	Challenges to incorporating DT in STEM lesson plans
“We chose the content because it’s a problem we encounter in daily life”	Real-life relevance of content	Topic selection rationale	Pedagogical reasoning in DT-oriented lesson plan
“We selected the topic because it was already in the curriculum and could be connected to daily life easily.”	Curriculum-driven content selection	Topic selection rationale	Pedagogical reasoning in DT-oriented lesson design
“We expect students to think creatively, be curious, use imagination, and enhance communication and cooperation skills.”	Anticipated development of 21st-century skills	Expected learning outcomes	Perceived instructional value of DT-based STEM lessons
“I think students would learn better because they are actively involved in producing something, not just solving problems.”	Learning through active engagement	Perceived learning benefits	Perceived instructional value of DT-based STEM lessons

To ensure interpretive transparency, the coding process was guided by a formal codebook specifying code definitions and operational boundaries. For example, the theme Challenges to Incorporating DT included the codes Planning and Discipline Alignment and Resources and Timing. The former was defined as statements describing difficulties in making the mathematics component explicit while simultaneously addressing science or engineering requirements, as illustrated by PMT 25’s statement that “The most challenging part was aligning the design with other disciplines and finding suitable learning outcomes.” The latter was defined as statements identifying time or material constraints that hindered the integration of DT and the meeting of the requirements of the national mathematics curriculum. This was reflected in PMT 16’s observation: “I think design is necessary, but it should not take too much time because there is a program that needs to be covered.” These code definitions and exemplars provided a systematic basis for interpreting participants’ responses and linking codes to higher-order themes.

To assess inter-rater reliability, the second researcher independently coded a randomly selected 25% sample of the questionnaire data (representing 14 of the 55 participants across all eight open-ended prompts), yielding an initial inter-rater agreement of 90%. In accordance with established qualitative research standards, this pre-discussion metric (90%) serves as the formal measure of inter-rater reliability, demonstrating strong initial coding consistency among independent analysts. Percentage agreement was selected as the primary metric for inter-rater consistency based on the nature of inductive, open-ended qualitative coding. Unlike closed, categorical survey coding, qualitative thematic analysis involves extracting emergent meaning units across an extensive, multi-level codebook. Consistent with Cole’s [69] recommendation, the researchers subsequently conducted a reconciliation session in which each point of disagreement was examined and discussed. Discrepancies were resolved by consensus, and code definitions were refined as necessary until complete agreement was reached. This iterative validation process strengthened the consistency and credibility of the analysis. The findings from lesson plan analyses and questionnaire data were compared and integrated in the interpretation to identify convergences and divergences between articulated beliefs and enacted design practices, thereby enhancing the study’s explanatory power.

The questionnaires were analyzed using an inductive approach rather than being guided by a predefined analytical framework. This decision was made to prioritize participants’ perspectives and to allow a comprehensive representation of PMTs’ experiences to emerge directly from the data. An inductive strategy was particularly appropriate for capturing variation in individual interpretations and experiences. Moreover, the inductive analysis enabled the identification of emergent themes that were not anticipated at the outset of the study. For instance, PMTs’ difficulties in aligning DT phases with instructional objectives and in selecting appropriate instructional materials emerged as salient issues in the data, which may have been constrained or overlooked within a strictly deductive framework.

Analysis was not conducted in isolation; instead, findings from the two embedded units were triangulated to support case-level interpretation. Frequencies from the group lesson plans were mapped against the emergent themes from individual questionnaires. This integrated analytical strategy enabled the researchers to determine whether missing design phases resulted from conceptual misunderstanding or from external factors reported by individuals. Consequently, the overarching case is interpreted through the lens of situated learning, focusing on how the participants navigated the transition from theoretical STEM knowledge to interdisciplinary instructional design.

To enhance the rigor and trustworthiness of the analysis, we employed methodological triangulation by examining group-level lesson plans alongside individually articulated reflections and assessing inter-coder agreement. This embedded single-case design enabled us to examine how DT phases were operationalized in shared lesson plans and to capture variation in participants’ individual interpretations within those contexts. The high inter-rater agreement, followed by consensus during questionnaire analysis, further strengthened the credibility and consistency of the findings. This systematic coding process, consistent with Cole [69], provided an audit trail linking the raw data to the final themes and supported the identification of the uneven implementation of the Empathy and Test phases as a consistent phenomenon across data sources.

## Results

### Pre-service mathematics teachers’ DT-oriented lesson plans

The first research question explored how PMTs integrated DT into their STEM lesson plans, using the Kim and Bolger [61] framework for analysis. The following Tables 5–7 present the distribution of STEM features and DT phases across the lesson plans, as well as insights into the integration of mathematics and DT.

**Table 5:** Frequencies and percentages of STEM integration features identified across collaborative lesson plans.

Features	STEM Features	Number of Lesson Plans	Percentages
Applicable Level for Students	The S, T, and E topics apply to students’ levels. (Using the textbook for the relevant grade level).	n=13	54.2%
Subject Interdependence	The S, T, and E content facilitates a better understanding of the M topic.	n=15	62.5%
Creative Application	Students are encouraged to draw innovative or original ties to the real world by including science, technology, and engineering in their studies.	n=18	75%
Math-focused	T, E, and S are integrated into math-related tasks, with M being the focus.	n=18	75%
Essential STE Inclusion	The mathematics topic effectively incorporates more than two S, T, and E concepts.	n=14	58.3%
Differentiating T from E	The lesson is designed to aid students in understanding T and E.	n=10	41.7%

N = 24 lesson plans. Percentages have been rounded to one decimal place.

**Table 6:** Implementation frequencies and percentages of DT phases in lesson plans.

DT Phases	Number of Lesson plans	Percentages
Empathy	n=5	20.8%
Define	n=23	95.8%
Ideate	n=20	83.3%
Prototype	n=22	91.7%
Test	n=13	54.2%

N = 24 lesson plans. Percentages have been rounded to one decimal place.

**Table 7:** Distribution of lesson plans by number of integrated DT phases.

High-level lesson plan DT score	Number of Lesson Plans	Percentages
5	n=3	12.5%
4	n=12	50%
3	n=5	20.8%
2	n=2	8.3%
1	n=1	4.2%
0	n=1	4.2%

N = 24 lesson plans. Percentages have been rounded to one decimal place.

Table 5 reveals that PMTs most frequently used the Creative Application feature (18 out of 24 lesson plans), reflecting a strong focus on engaging students with real-world contexts through STEM. The Math-focused feature, where mathematics is central, appeared in 18 plans, indicating that PMTs prioritized mathematics within the STEM framework. The Subject Interdependence feature was present in 15 lesson plans, suggesting that some PMTs sought to enhance students’ understanding of mathematics by linking it with science, technology, and engineering. On the other hand, **Essential** STE Inclusion appeared in 14 plans, demonstrating a moderate integration of multiple STEM concepts. The Applicable Level for Students feature, found in 13 plans, and Differentiating T from E in 10 plans, highlight areas where alignment with student needs and clarity in distinguishing between technology and engineering could be improved.

Most of the lesson plans (n = 18) effectively emphasized mathematics. For instance, the 24th lesson plan (Table 2) focused on periscope design, using geometric concepts like angles, reflections, and measurements to create a functional periscope. Thus, this lesson plan allowed students to apply their math knowledge in a practical context. Likewise, the fifth lesson plan focused on environmental awareness and recycling, with an emphasis on designing appealing recycling bins. It emphasized the surface development of a cylinder.

The most frequently implemented DT phases were Define, Ideate, and Prototype. In contrast, the Empathy and Test phases were the least incorporated, reflecting a potential gap in understanding of the DT process.

As detailed in Table 6, PMTs’ operationalization of DT varied considerably across the five phases rather than following a consistent pattern of decline. The Define (95.8%), Ideate (83.3%), and Prototype (91.7%) phases formed the dominant procedural core. Examination of the lesson plans further revealed patterns in PMTs’ pedagogical reasoning regarding DT integration. The prominence of these three phases suggests that PMTs tended to conceptualize DT primarily as a linear problem-solving process oriented toward product development, rather than as an iterative, user-centered design cycle. In many plans, problem definition was largely teacher-directed and followed by idea generation and construction activities, while opportunities for reflection, feedback, and iterative refinement were comparatively limited.

The data demonstrate a clear distinction between the particularly limited inclusion of Empathy and the comparatively moderate implementation of Test. Empathy (20.8%) was by far the least-represented phase, largely omitted by four-fifths of the cohort. In most lesson plans, PMTs bypassed user-centered inquiry. The limited incorporation of the Empathy phase indicates that user-centered design principles were not fully operationalized in mathematics-focused lesson planning. In most cases, the “user” was implicitly assumed or replaced by curricular objectives, suggesting a tension between DT’s emphasis on understanding user needs and PMTs’ content-driven views of mathematics instruction. Unlike Empathy, the Test (54.2%) phase was incorporated in more than half of the lesson plans. However, it still showed a notable decrease relative to the Define, Ideate, and Prototype phases. The underrepresentation of the Test phase points to a constrained understanding of iteration, with prototypes frequently treated as final products rather than as solutions to be evaluated and improved.

Based on the inclusion of all DT phases (see Table 7), the scores show that only three lesson plans earned the highest score of 5 (incorporating all DT phases). Twelve plans scored 4, indicating a substantial DT integration, but some phases were missing. Notably, one lesson plan received a score of 0, suggesting no DT phases were integrated.

As shown in Table 7, a score of 5 indicates that all five phases achieved robust mathematical integration (12.5%). The single plan scoring 0 was deficient not only because DT phases were largely unformatted, but because mathematics remained entirely isolated from the design tasks. The integration of mathematics and DT varied among the lesson plans. As an example, the 2nd lesson plan (Table 2), which focused on kite design, incorporated all DT phases: Empathy (exploring kites and gathering user feedback), Define (articulating the core problem), Ideate (brainstorming and sketching designs), Prototype (creating physical models), and Test (evaluating and improving the kites). This approach allowed students to engage in mathematical (e.g., area and design concepts) and DT processes. In contrast, the 10th lesson plan, which involved making a thermometer, only included the Define, Ideate, and Prototype phases. It lacked the Empathy phase (no consideration of user needs and no quantitative or mathematical problem-scoping criteria) and either omitted the Test phase or included a physical test (e.g., whether a structure stands), but did so qualitatively, without systematic data collection, measurement, or mathematical optimization.

Comparisons between high- and low-scoring lesson plans further showed that stronger DT integration was associated with using mathematics as an analytical tool across multiple DT phases, particularly during testing and refinement. In contrast, lower-scoring plans tended to confine mathematics to the Define phase, positioning it as background knowledge rather than as a resource for evaluating and optimizing designs. Overall, these findings suggest that PMTs’ partial implementation of DT phases reflects not only practical constraints but also deeper challenges in reconciling iterative design processes with curricular expectations and established mathematics teaching practices.

### Pre-service mathematics teachers’ experiences in planning DT-oriented lesson plans

The study's second research question explored how PMTs select DT-oriented topics and the challenges they face in planning. The following sub-sections present participants’ experiences in these areas.

#### Deciding the DT topic for a lesson.

A key theme in the PMTs’ experiences was the Selection of a DT topic for the STEM lesson plan. Fig 2 outlines the categories, codes, and frequencies associated with this decision.

**Fig 2:**
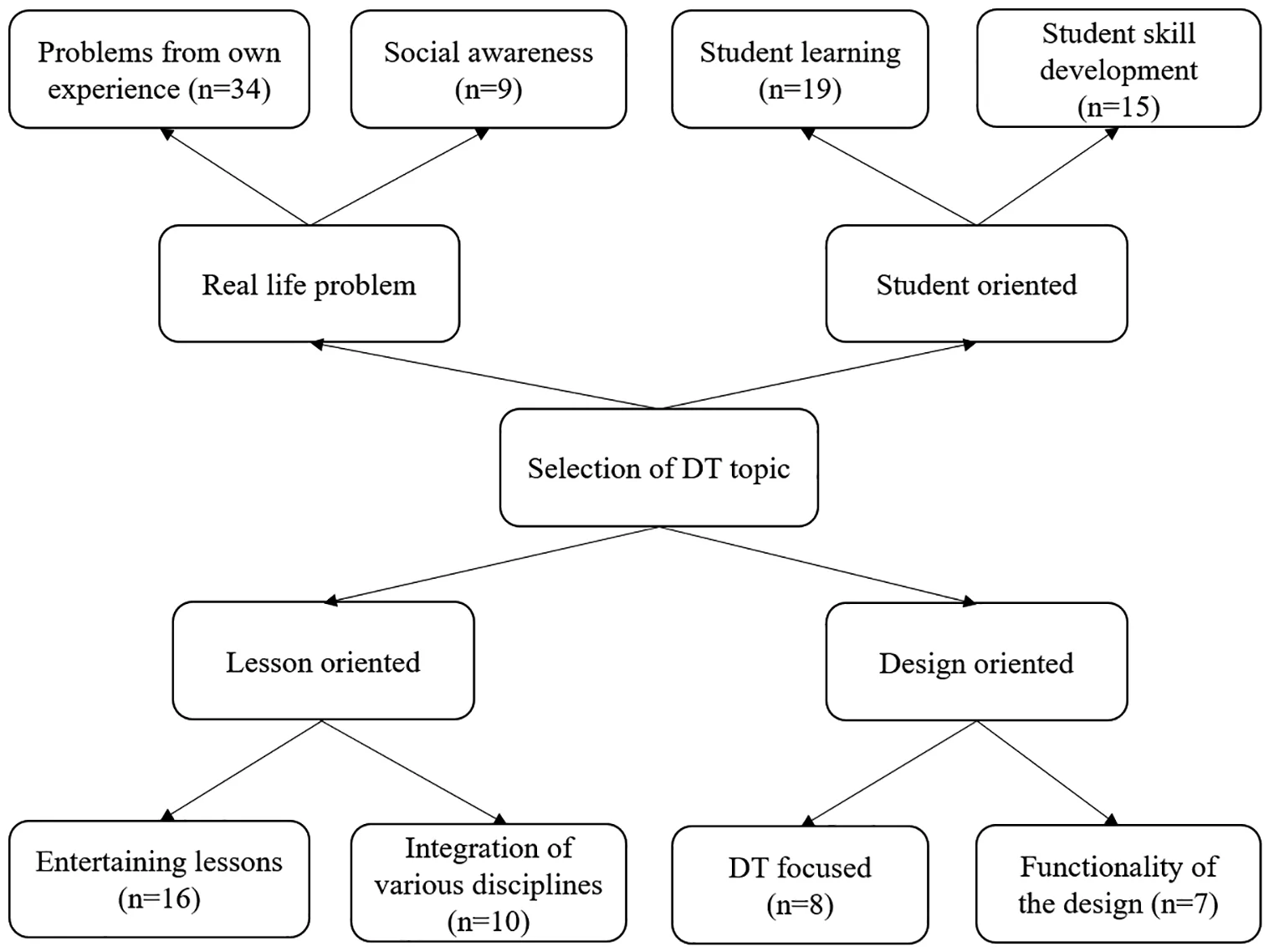
Thematic distribution of PMTs’ pedagogical rationales for topic selection in DT-oriented STEM lesson plans.

Fig 2 highlights four major themes in selecting a DT topic: Real-life Problem, Student-oriented, Lesson-oriented, and Design-oriented. The most frequent codes were ‘problems from own experience’ (n = 34) and ‘student learning’ (n = 19). PMTs were more focused on addressing real-life problems and ensuring lessons were student-oriented. Below are excerpts that illustrate these themes:

*We chose the content because it's a problem we encounter in daily life. (PMT 9)*

*We aimed to develop children's imagination by helping them better understand geometric objects. (PMT 38)*

These excerpts suggest that PMTs often prioritized real-life relevance and student engagement in choosing their topics. While DT was considered, the emphasis on student-centered learning and the problem's real-world applicability was prioritized.

#### Challenges in incorporating DT in lesson plans.

The second theme from participants’ experiences focuses on the challenges in preparing DT lesson plans. Fig 3 outlines the categories, codes, and frequencies associated with these difficulties.

**Fig 3:**
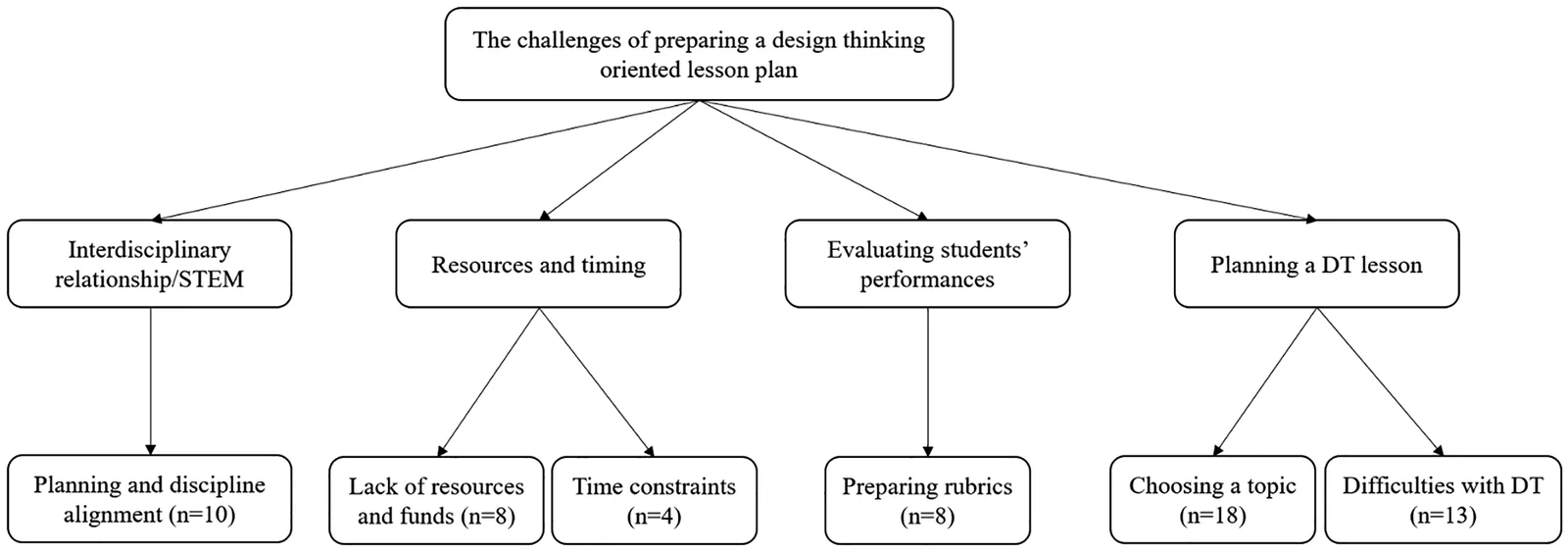
Emergent categories and code frequencies of challenges encountered by PMTs during DT lesson planning.

Four key themes emerged: Interdisciplinary Relationship/STEM, Resources and Timing, Evaluating Students’ Performances, and Planning a DT Lesson. The most frequent codes were ‘Choosing a topic’ (n = 18), ‘Difficulties with DT’ (n = 13), and ‘Planning and Discipline Alignment’ (n = 10). The biggest challenges identified were selecting appropriate topics and addressing difficulties integrating DT. Below are excerpts reflecting these struggles:

*The most challenging part was aligning the design with other disciplines and finding suitable learning outcomes. (PMT 25)*

*It was challenging to find up-to-date questions for each DT step and to evaluate the originality of students’ designs. (PMT 41)*

*We struggled with how to provide and limit materials for student designs. (PMT 15)*

These comments reveal several barriers to successfully incorporating DT: difficulties in aligning mathematics with other STEM disciplines, challenges in crafting effective DT questions, and struggles with managing materials and assessing student creativity. While PMTs acknowledged the value of integrating disciplines through DT, they found it difficult to do so meaningfully without compromising mathematical rigor.

Taken together, the findings suggest that PMTs’ decisions about DT-oriented topics and their reported planning challenges are closely interconnected and reflect deeper tensions in DT integration. While PMTs predominantly selected topics grounded in real-life problems and student engagement, these choices were often guided by familiarity and perceived relevance rather than by a clear articulation of user needs or iterative design goals. This tendency helps explain why difficulties emerged in later planning stages, particularly in aligning mathematics with other STEM disciplines and structuring DT phases coherently. The emphasis on authenticity and student-centeredness, although pedagogically valuable, did not consistently translate into robust interdisciplinary or iterative design practices. Instead, DT was frequently treated as an add-on to existing lesson structures, making it challenging for PMTs to balance creative design activities with curricular demands and mathematical rigor. These patterns indicate that PMTs’ challenges were not merely practical but also rooted in evolving conceptions of DT as a pedagogical framework in mathematics-focused STEM instruction.

### Pre-service mathematics teachers’ views on DT in STEM lessons

The third research question explored PMTs’ views on integrating DT into STEM lessons. Two key themes emerged: Expected outcomes and benefits of DT-oriented lessons, and DT's role, alignment, and impact in mathematics education.

#### Expected outcomes and benefits of DT-oriented lessons.

When asked about expected learning outcomes, PMTs emphasized developing 21st-century skills and mathematical exploration through hands-on design processes (Table 8).

**Table 8:** PMTs’ anticipated student competencies and learning outcomes from DT-oriented STEM lessons.

Expected skills that students will employ during DT activities	Number of responses	Percentages	Expected learning outcomes	Number of responses	Percentages
Modelling	n=2	3.6%	Contextual learning	n=8	14.6%
Social responsibility and awareness	n=16	29.1%	Employing all DT phases	n=6	10.9%
Interdisciplinary association	n=9	16.4%	Exploration/Curiosity	n=8	14.6%
Conceptual thinking	n=7	12.7%	Learning math by design/experience	n=11	20%
Positive learning environment, learning with fun	n=3	5.5%	Flexible/genuine learning	n=8	14.6%
Innovative thinking	n=4	7.3%	Applying 21st-century skills	n=5	9.1%
21st-century skills	n=35	63.6%	Sense of responsibility/awareness by designing	n=1	1.8%
Design	n=15	27.3%	Performance and process-based learning	n=21	38.2%
Noticing math in real life	n=27	49.1%			

*N* = 55 respondents. Percentages have been rounded to one decimal place.

As illustrated in Table 8, the primary skills anticipated from students were 21st-century skills and the ability to notice mathematics in real-life contexts. Similarly, the most anticipated learning outcomes included performance-based and process-oriented learning, as well as the acquisition of mathematical knowledge through design and experiential methods. Below are some participants’ views on these outcomes.

*We expect students to think creatively, be curious, use imagination, and enhance communication and cooperation skills. (PMT 22)*

*Students are expected to connect mathematics with their daily experiences. (PMT 34)*

*I hope to observe and evaluate students’ learning using a rubric and peer assessment. (PMT 40)*

Additionally, participants reflected on the benefits of planning and creating STEM lesson plans focused on mathematics and the DT framework. Their views, illustrated in Fig 4, fall into four major categories: Student Aspect, Teacher Aspect, Math Aspect, and Course Aspect.

**Fig 4:**
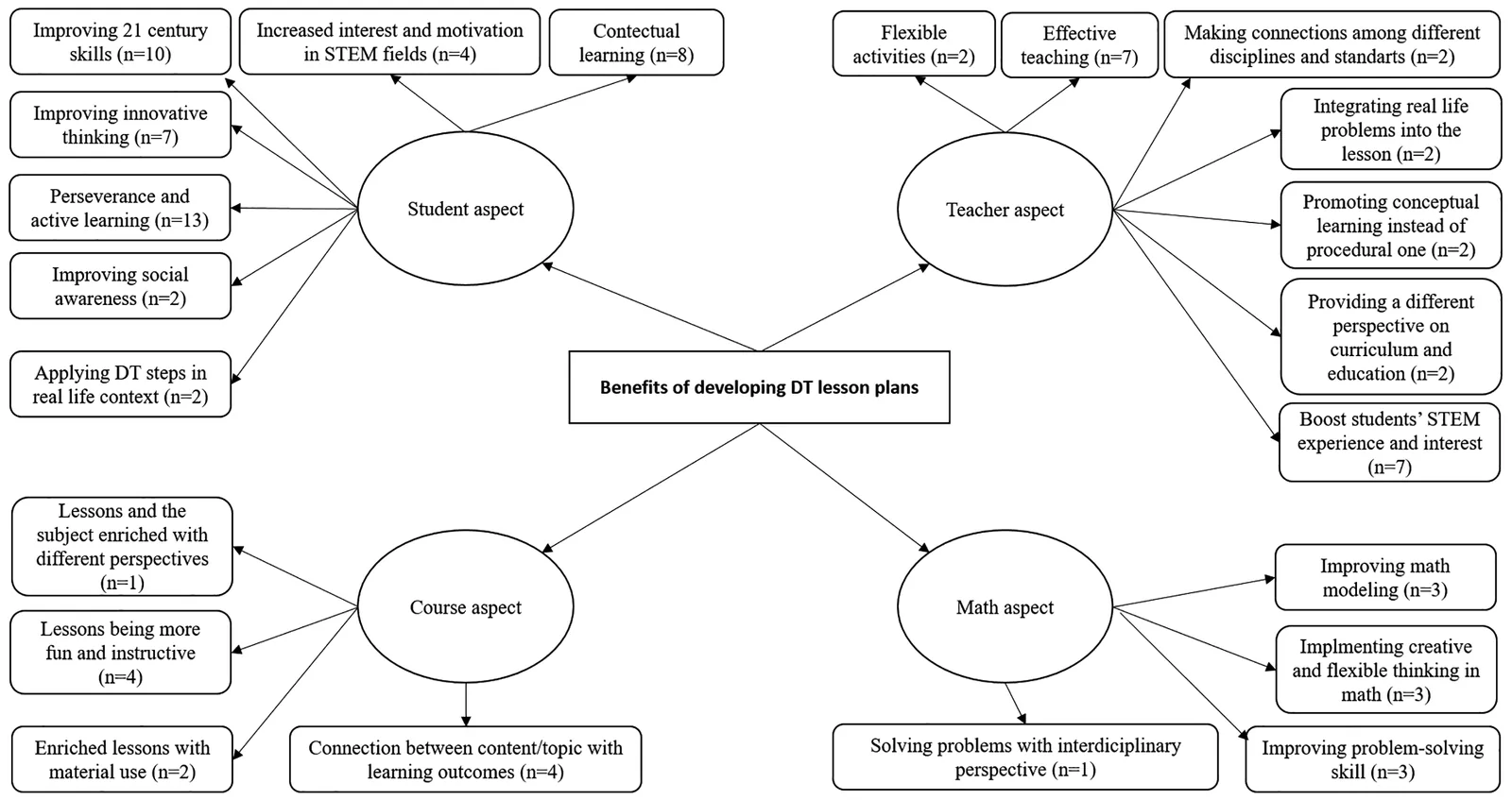
Multidimensional benefits of designing DT-oriented STEM lessons as perceived by PMTs.

Under the Student Aspect, the most common benefits identified were perseverance and active learning (n = 13), improving 21st-century skills (n = 10), and contextual learning (n = 8). As some participants noted:

*Students learn deeply through active involvement in all stages, making the information memorable and understandable. (PMT 19)*

*Students will become active learners, gain new perspectives, and develop empathy, creativity, problem-solving skills, and group work skills. (PMT 34)*

Regarding the Teacher Aspect, PMTs noted benefits such as effective teaching (n = 7) and boosting students’ STEM experience and interest (n = 7). For example:

*I've gained STEM experience and will be adept at preparing DT-oriented lesson plans in the future. (PMT 39)*

*I learned to develop design-oriented projects and plan each step effectively. I will teach my students DT and provide STEM experiences professionally. (PMT 44)*

Finally, for the Course and Math Aspects, common observations included that lessons might be more fun and instructive (n = 4) and foster creative and flexible thinking in mathematics (n = 3):

*The positive side was seeing that we could have fun in the course. (PMT 35)*

*I think DT contributes to being more original, flexible, and creative in math subjects. (PMT 21)*

PMTs anticipate that incorporating DT into STEM lessons will develop essential skills, such as 21st-century competencies and the ability to connect mathematics to real-life contexts. They expect students to engage in performance-based and process-oriented learning, fostering creativity, curiosity, and active participation. Teachers believe that planning DT-oriented lesson plans will enhance student learning and improve their teaching effectiveness by integrating content into real-world applications. Additionally, these lesson plans are expected to make mathematics instruction more engaging, innovative, and flexible, ultimately benefiting students and teachers by fostering deeper understanding and professional growth.

#### DT's role, alignment, and impact in mathematics education.

This section presents PMTs’ views on the role of DT in the mathematics curriculum. Most participants (n = 43) believed that mathematics teachers should be equipped to develop DT-oriented lesson plans (Table 9), though a few (n = 7) expressed that this competency may only be essential for some. When asked whether DT aligns with the mathematics curriculum, responses were mixed: while 28 participants said yes, others noted partial alignment (n = 12) or disagreed entirely (n = 8).

**Table 9:** PMTs’ perceptions of required teacher competencies and national curriculum alignment for DT in mathematics.

Questions Posed to PMTs	Response Categories	Frequencies	Percentages
Whether Mathematics teachers should have skills for developing DT-oriented lesson plans.	Yes	n=43	78.2%
	No	n=1	1.8%
	Some teachers should have	n=7	12.7%
	No response	n=4	7.3%
Compliance of DT with the middle school mathematics curriculum	Yes	n=28	50.9%
	No	n=8	14.6%
	Partially	n=12	21.8%
	No response	n=7	12.7%

*N* = 55 respondents. Percentages have been rounded to one decimal place.

Below are some participants’ views on these outcomes:

*Active student engagement promotes lasting learning. Effective DT lesson plans ensure this engagement and productive use of time, so teachers need to be skilled in planning DT lessons. (PMT 21)*

*I think design is necessary, but it should not take too much time because there is a program that needs to be covered, an annual plan. (PMT 16)*

*It meets the curriculum needs but may waste time on assessment and be challenging in exam-focused grades. (PMT 8)*

PMT 21 emphasized that DT promotes active student engagement, leading to permanent learning, and supports the productive use of classroom time. In contrast, PMT 16 raised concerns about the time required for the design process, which could interfere with covering the required curriculum. Other participants, such as PMT 8, doubted DT's practicality in assessment-driven contexts and a curriculum with heavy content demands.

Table 10 captures both the advantages and disadvantages as perceived by the participants. On the positive side, they cited improved engagement, contextual learning, and development of key skills. However, concerns about time consumption (n = 30) and logistical challenges (e.g., materials, classroom size) were prevalent.

**Table 10:** Perceived pedagogical affordances (advantages) and practical constraints (disadvantages) of integrating DT into mathematics instruction.

Categories	Response Categories	Number of responses	Percentages
Advantages	Mathematical modeling	n=12	21.8%
	Contextual learning	n=19	34.6%
	Active engagement in learning and having fun in courses	n=26	47.3%
	Students may have new learning perspectives and thinking styles	n=8	14.6%
	Boost students’ ability and motivation to design	n=7	12.8%
	Develop 21st-century skills	n=15	27.3%
	Develop social awareness and skills	n=4	7.3%
	Interdisciplinary association and gaining various learning outcomes	n=8	14.6%
Disadvantages	Time-consuming	n=30	54.6%
	Workload for teachers	n=6	10.9%
	Difficulties with classroom management and implementation of DT in crowded classrooms	n=4	7.3%
	Disagreements within and between groups	n=4	7.3%
	Students may find them time-consuming and boring	n=5	9.1%
	Students may struggle in the design process	n=3	5.6%
	Difficulties in supplying materials, lack of suitable environment	n=10	18.2%
	DT may not apply to all math subjects	n=7	12.8%
	There is no disadvantage	n=7	12.8%

*N* = 55 respondents. Percentages have been rounded to one decimal place.

The most frequently mentioned advantages include active engagement in learning and making math fun (n = 26), promoting contextual learning (n = 19), developing 21st-century skills (n = 15), and facilitating mathematical modeling (n = 12).

*It creates a learning environment where the student is active, and thus learning is more permanent. (PMT 20)*

*I think DT in education fosters skills such as responsibility, creativity, originality, cooperation, problem-solving, critical thinking, adaptability, entrepreneurship, curiosity, and imagination. (PMT 22)*

For instance, PMT 20 highlighted that DT fosters an engaging, active learning environment, thereby contributing to long-term knowledge retention. Similarly, PMT 22 noted that DT helps students develop essential skills, such as creativity, critical thinking, and cooperation. However, several disadvantages were noted, with time consumption being the most frequently cited concern (n = 30). Other challenges include providing necessary materials and suitable environments (n = 10).

*A preliminary plan is needed for materials, and implementation will take more time and cost more than standard lectures. (PMT 22)*

*Its implementation in crowded classrooms can be confusing, time-consuming, and costly. (PMT 45)*

PMT 22 further pointed out that DT necessitates additional resources and planning, which can be more demanding than traditional teaching methods. PMT 45 added that the complexities of managing DT in crowded classrooms could lead to confusion and inefficiency.

In summary, PMTs recognized the value of DT for fostering student engagement, creativity, and interdisciplinary thinking. They anticipated that DT-oriented lessons could make mathematics more relevant, experiential, and enjoyable. However, their interpretations of DT were often incomplete, with less frequent integration of the critical Test phase. Addressing the third research question, these findings reveal that PMTs generally view the integration of design thinking and mathematics in STEM education as pedagogically valuable yet contextually constrained. PMTs articulated strong beliefs that DT-oriented STEM lessons can enhance students’ engagement, support the development of 21st-century skills, and enable meaningful connections between mathematics and real-life contexts through experiential and performance-based learning. At the same time, their views reflect an awareness of practical tensions, particularly regarding time constraints, curricular coverage, assessment pressures, and resource availability. While most participants endorsed the idea that mathematics teachers should be capable of designing DT-oriented lessons, their mixed perceptions of curricular alignment and feasibility suggest that PMTs conceptualize DT not as a universally applicable approach, but as a powerful yet situational pedagogical tool. Together, these views indicate that PMTs value DT as a means of enriching mathematics learning, while simultaneously negotiating its role within the structural realities of school mathematics.

## Discussion

This study investigated how PMTs integrate DT and mathematics into STEM lesson planning, the challenges they face, and their views on the educational value of DT. The findings illuminate PMTs’ pedagogical strategies and evolving conceptions of DT in mathematics education, aligning with calls for interdisciplinary approaches that maintain mathematical rigor [2,6,33]. The findings suggest that DT serves as a foundational mechanism for linking disciplinary knowledge in integrated STEM contexts. Rather than operating as a separate subject, DT provides the foundational linking processes required to navigate complex, real-world problems that do not exist in disciplinary isolation. PMTs’ DT lesson topics were strongly influenced by the design thinking course’s emphasis on creativity, student-centered learning, and real-life problem-solving, highlighting the role of coursework as a pedagogical scaffold for initial engagement in DT-oriented STEM planning. PMTs also perceived DT lesson planning as beneficial for promoting active learning, 21st-century skills, creativity, flexible mathematical thinking, and teaching effectiveness. These perceptions reflected intentions to connect mathematics with authentic contexts and engage students in process-oriented learning, positioning DT as a meaningful approach for both student learning and teacher development.

Most lesson plans (18 of 24, 75%) incorporated substantial mathematical content into DT-oriented STEM activities. This finding is relevant to concerns in the literature that mathematics may be marginalized in integrated STEM instruction [7,8,12]. The plans incorporated mathematical modeling, measurement, and geometric reasoning into design tasks, consistent with arguments that mathematics should play an integral role in interdisciplinary STEM learning [30,43]. In these plans, mathematics was used to support design decisions concerning factors such as cost, stability, and functionality, rather than serving solely as contextual background. These findings suggest that DT-oriented lesson planning can provide opportunities for positioning mathematics as an integral component of interdisciplinary problem-solving. However, the extent to which this potential is realized depends on how explicitly mathematical reasoning is integrated into design decisions in the planned activities.

The frequent inclusion of Creative Application and Subject Interdependence features suggests that PMTs incorporated opportunities to apply knowledge across disciplines and to connect mathematical concepts with design tasks. This finding is consistent with the literature advocating real-world, student-centered mathematics instruction [13,18,65]. However, the uneven representation of DT phases, particularly the limited treatment of Empathy and Test, suggests that the iterative and user-centered dimensions of DT were not consistently reflected in the lesson plans. Only three of the 24 lesson plans incorporated all five DT phases, indicating that comprehensive representation of the DT process was uncommon. This pattern may suggest that PMTs placed greater emphasis on developing design solutions than on systematically incorporating user needs and testing into the planned activities. These findings are consistent with previous research highlighting challenges novice educators may encounter when implementing DT as an integrated process [14,61].

While prior literature has documented novice designers’ difficulties with the non-linear nature of design cycles, our findings provide a more nuanced picture. The limited inclusion of the Empathy phase (20.8%) suggests that PMTs had difficulty framing mathematics-related problems in human-centered, socially situated terms and often relied on teacher-assigned problems. Although the Test phase appeared in 13 of 24 lesson plans (54.2%), indicating some attention to evaluation, its lower prevalence than Define (95.8%) and Prototype (91.7%) suggests that testing was more vulnerable to omission or simplification in instructional planning.

The less frequent integration of the Test phase into lesson plans suggests fewer planned opportunities for iterative evaluation and reflective mathematical reasoning, which are central to effective STEM learning [11,12]. This pattern can be interpreted through situated cognition theory: Although PMTs selected authentic contexts for their design tasks, the lesson plans did not consistently demonstrate how disciplinary knowledge and tools would be coordinated to support iterative design. From the perspective of English’s [11] framework, the limited representation of the Test phase may also constrain opportunities for learning innovation by reducing the emphasis on evaluating and refining design solutions. Although student learning was not directly measured through classroom enactment, the lesson plans suggest that prototypes were sometimes treated as final products rather than as models to be tested and revised. This demonstrates that a potential challenge for PMTs is not merely remembering to include a testing step, but conceptualizing test results as empirical mathematical data for design optimization. Overall, DT appeared in some plans as a means of product creation rather than as an iterative process of design refinement. These findings highlight the importance of teacher education programs explicitly addressing iterative design, reflective reasoning, and mathematical modeling [21].

Furthermore, the findings offer a nuanced comparison with Boakes [64]. While Boakes reported that middle school girls initially experienced discomfort with the open-ended nature of DT, they eventually used testing methods (e.g., placing heavy reams of paper on structures to assess stability) to refine their designs. The lesson plans in the present study incorporated iterative testing and refinement less consistently. This difference may reflect the distinct roles of the participants. The students in Boakes’s study engaged in design activities, whereas the PMTs in the present study were planning instructional activities for others. The findings raise questions about how prospective teachers conceptualize and plan for the cyclical nature of DT within the constraints of school curricula. This issue should also be considered alongside the practical challenges reported by participants, including time constraints (n = 30) and difficulties with materials (n = 10), which may have influenced their plans for implementing the design cycle. Taken together, these findings highlight the importance of teacher education programs that address not only the phases of DT but also the integration of testing, reflection, and iterative refinement into feasible, mathematics-focused STEM lessons.

PMTs’ topic selection strategies, often guided by student interests and real-life relevance, reflected learner-centered approaches consistent with prior literature [15]. At the same time, PMTs reported challenges in aligning DT phases with curricular objectives, selecting appropriate materials, and designing assessments. These difficulties mirror broader issues identified in studies of interdisciplinary planning and DT integration [7]. PMTs particularly struggled to translate abstract DT principles such as empathy and iteration into practical pedagogical strategies, supporting calls for stronger scaffolding in teacher education programs [21,24].

A major strength of this study lies in the triangulation of lesson plan analyses and questionnaire responses. The integration of these data sources strengthened the interpretation of the findings by connecting intended instructional designs with participants’ reflections and reported challenges. For example, the frequent inclusion of Creative Application (n = 18) and Math-focused integration (n = 18) in lesson plans corresponded with questionnaire findings emphasizing Real-life Problems (n = 34) as the primary factor influencing topic selection. Similarly, the limited representation of the Test phase (n = 13) in the lesson plans can be considered alongside participants’ reports of time constraints (n = 30) and material-related difficulties (n = 10). Taken together, these findings suggest that the challenges associated with incorporating iterative refinement may involve both pedagogical considerations and practical constraints. However, the extent to which these constraints directly influenced the representation of the Test phase cannot be established from the available data alone.

From a teacher learning perspective, the findings can be interpreted through situated learning and reflective practice theories, which emphasize the role of authentic participation and reflection in professional learning [27,28].

The uneven representation of DT phases, particularly Empathy and Test, suggests that translating DT principles into comprehensive instructional plans may be challenging for pre-service mathematics teachers. These patterns may reflect the limited opportunities available within a single course for repeated cycles of design, implementation, reflection, and refinement, although the present study cannot directly establish this relationship. Although participation in a DT-focused course provided an important introduction to the approach, further opportunities to develop, enact, evaluate, and revise DT-oriented lessons may help PMTs develop a more integrated understanding of DT as an iterative pedagogical framework. From this perspective, the challenges identified point to broader considerations for teacher education, including the need to provide structured opportunities for authentic practice and reflection, rather than being interpreted solely as individual shortcomings.

The challenges reported by PMTs are also relevant to those discussed in the literature on other ambitious instructional approaches, including mathematical modeling, inquiry-based learning, and cognitively demanding tasks [73,74]. As reported in studies of modeling-based instruction, the findings suggest a potential tension between designing open-ended learning experiences and addressing curricular and assessment expectations. This comparison situates the challenges of DT integration within broader discussions of implementing student-centered, cognitively demanding mathematics instruction.

Despite these challenges, PMTs expressed positive views about the pedagogical value of DT, particularly its capacity to support creativity, collaboration, and problem-solving associated with 21st-century learning [6,13]. Participants perceived that DT-based activities could make abstract mathematical concepts more tangible through modeling, construction, and measurement. Lesson plan examples involving periscopes and kites illustrated opportunities to incorporate geometry and spatial reasoning into meaningful, hands-on design activities. These findings are consistent with research highlighting the potential of DT to support application-oriented learning and connections between theory and practice [6,15,18,25]. At the same time, PMTs identified barriers to integrating DT, including time constraints, curricular pacing, and assessment pressures, echoing challenges reported in previous studies [16,19,56]. Although DT emphasizes exploration and iterative refinement, incorporating these processes into instructional plans may be challenging within educational settings that prioritize curriculum coverage and standardized assessment.

## Conclusion

This study examined the integration of DT into mathematics-focused STEM lesson planning among PMTs. The findings indicate that many PMTs incorporated substantial mathematical content into interdisciplinary design tasks, suggesting that DT-oriented lesson planning can help position mathematics as an integral component of STEM education. By examining group-developed lesson plans alongside individual questionnaire responses, the study connected the representation of mathematical content and DT phases with participants’ reflections and reported challenges. However, the representation of DT phases was uneven. Although the Define, Ideate, and Prototype phases appeared in the majority of lesson plans, Empathy was included in only 20.8% of plans, while Test appeared in 54.2%. Only three of the 24 lesson plans incorporated all five phases. These findings suggest that although PMTs frequently planned activities focused on developing design solutions, the user-centered and iterative dimensions of DT were less consistently represented. In some plans, DT appeared to emphasize product creation more than the systematic testing and refinement of design solutions through mathematical reasoning.

### Implications for mathematics education

The findings have implications for mathematics education, particularly for the design of mathematics-focused STEM instruction. When iterative testing is not explicitly incorporated into lesson plans, opportunities for students to use mathematical modeling to evaluate and refine design solutions may be limited. Future research should build on these qualitative findings by conducting quasi-experimental and longitudinal classroom studies that directly examine student learning. Such studies could investigate whether systematically incorporating the Empathy and Test phases into DT-oriented STEM lessons supports students’ adaptive problem-solving and mathematical competence.

The findings also highlight the need for teacher education programs to provide more explicit scaffolding for mathematics-rich, iterative, DT-oriented STEM instruction. Although PMTs emphasized real-world relevance and student-centered learning, the limited representation of the Empathy and Test phases suggests that the user-centered and iterative dimensions of DT were not consistently reflected in their lesson plans. Teacher education programs should therefore provide opportunities for PMTs to experience and practice DT as a cyclical process in which mathematical reasoning informs the testing, evaluation, and refinement of design solutions. Explicit modeling of DT-oriented lessons, interdisciplinary curriculum mapping, and assessment approaches that value creativity, design processes, and conceptual understanding may help PMTs integrate DT and mathematics more coherently [11,12,33,59,60,75].

To bridge the identified uneven representation of the Empathy and Test phases, teacher education programs may move beyond theoretical exposure and provide structured pedagogical scaffolding that mirrors the iterative nature of design. Specifically, to shift the Empathy phase from a teacher-directed objective to a user-centered inquiry, programs should use probing and clarifying questions as primary instructional tools. By training PMTs to ask, “What problem are you seeking to solve and why is it important to the user?” educators can prompt future teachers to transition from abstract topic selection to community-based modeling [69,75]. In the Empathy phase, students identify the person(s) they are designing for and the users’ roles and needs in the context [45]. This approach may help PMTs select locally relevant problems in which mathematical reasoning serves a meaningful purpose within an interdisciplinary STEM task, rather than merely functioning as a procedural requirement.

To strengthen the representation of the Test phase, teacher education programs could emphasize learning through iterative design and generative learning tasks [11]. Providing ongoing formative feedback during lesson planning could help PMTs specify how test data will be analyzed and used to evaluate and refine prototypes [61]. This approach would position testing not simply as the final step in the design process but as an opportunity to use mathematical evidence to inform subsequent design decisions and support adaptive problem-solving. In practice, PMTs could develop data tables, recording templates, or mathematical modeling guides for their lesson plans to document design performance against predefined criteria and constraints [76]. Such scaffolds may help PMTs make the relationship between testing, mathematical reasoning, and design refinement more explicit.

Finally, translating these recommendations into practice may be supported by providing exemplar DT-oriented STEM lessons and opportunities for interdisciplinary curriculum mapping. Kim and Bolger [61] emphasize the value of incorporating high-quality STEM examples into instructional materials to support teachers’ implementation of integrated STEM approaches. In addition, teacher education programs could organize collaborative design cycles in which PMTs engage with perspectives from different disciplines and explore how mathematical reasoning can be integrated with engineering constraints and design objectives [2]. Such experiences may help PMTs establish meaningful connections between mathematics and other STEM disciplines while ensuring that mathematical concepts and reasoning play an explicit and substantive role in addressing the design problem.

### Contributions of the study

This study contributes to the integrated STEM literature by providing empirical insight into how PMTs incorporate DT into mathematics-focused STEM lesson planning. The findings suggest that DT-oriented lesson planning can provide a framework for designing interdisciplinary tasks that go beyond routine problem-solving and create opportunities to apply mathematical reasoning in authentic design contexts. Although the study examined lesson plans and participants’ reflections rather than classroom enactment or student learning outcomes, the findings identify instructional design features that may support adaptive reasoning and the application of mathematical concepts. They also highlight challenges in reconciling the open-ended nature of DT with curricular expectations and practical constraints, including time constraints.

A key contribution of the study is its identification of uneven representation of the Empathy and Test phases in PMTs’ lesson plans. Although mathematical content was substantial in 18 of the 24 plans, the user-centered and iterative dimensions of DT were less consistently represented. In particular, the findings suggest that mathematics was more often incorporated into defining design problems and developing prototypes than explicitly positioned as a tool for evaluating test results and refining design solutions. By examining lesson plans alongside questionnaire responses, the study provides a more nuanced understanding of how mathematical integration and DT processes were represented in planned instruction and how these patterns related to participants’ reported challenges. This extends discussions of mathematics as an underrepresented or “silent” partner in STEM education by illustrating both the potential of DT-oriented planning to foreground mathematics and the challenges of sustaining mathematical reasoning throughout the design cycle. However, the findings do not establish that these patterns resulted solely from limited understanding of DT or assessment-driven curricular structures.

The findings also have implications for teacher preparation and research. Teacher education programs could provide structured opportunities for PMTs to develop, evaluate, and revise DT-oriented STEM lesson plans, with particular attention to how mathematical evidence informs design decisions during testing and refinement. Exemplars, interdisciplinary curriculum mapping, formative feedback, and explicit guidance on interpreting test data may help PMTs connect mathematical reasoning with the iterative nature of DT. Theoretically, the study extends discussions of DT and mathematics-focused STEM education by highlighting the importance of examining not only whether design phases appear in lesson plans but also how mathematical reasoning is intended to function within those phases.

### Limitations of the study

This study has several methodological limitations that should be acknowledged. First, the data were collected from a single cohort of 55 PMTs at a state university in Türkiye. The findings are therefore situated within a specific institutional and national educational framework and are not intended to be statistically generalizable to PMTs in other settings. Consistent with Yin’s [67] account of analytic generalization, the purpose of this case study was to relate the findings to relevant theoretical perspectives rather than to generalize statistically to a wider population. Accordingly, the identified patterns offer context-specific insights into how PMTs represent DT processes in interdisciplinary lesson planning, rather than a definitive model of how novice teachers generally approach DT integration. Second, participants were selected based on their senior status and engagement with the course. While this provided a relevant context for examining their development of interdisciplinary lesson plans, the sample may not represent PMTs at other stages of teacher preparation or those with different levels of engagement and prior experience.

Third, the author’s dual role as both the instructor and the researcher may have influenced participants’ responses and the interpretation of the data. Although participation was voluntary and non-evaluative, and the questionnaire took place after the course concluded, these measures cannot fully eliminate the possibility of social desirability bias or of the researcher’s position and expertise influencing the instructional context and analysis. As discussed by Boakes [64], researcher involvement in exploratory studies may shape the learning experiences and data generated, particularly when the researcher facilitates activities involving the approach under investigation. This possibility should be considered when interpreting the findings. Finally, the study relied on lesson-plan artifacts and self-reported questionnaire responses rather than observations of classroom enactment. The findings therefore reflect the instructional designs PMTs developed and their reported perspectives, not how the planned lessons would unfold in practice. As Kim and Bolger [61] suggest, the transition from integrated STEM lesson planning to classroom implementation may involve additional challenges arising from practical constraints and student responses.

### Recommendations

Future research could address the limitations of this study by examining DT-oriented STEM instruction across multiple institutions, cohorts, and educational contexts. Longitudinal studies incorporating classroom observations, student work, and measures of student learning could investigate how PMTs’ lesson-planning intentions translate into instructional practice and how integrating DT and mathematics relates to students’ mathematical learning and adaptive problem-solving. Further research could also compare different forms of pedagogical scaffolding to determine how they support the integration of mathematical reasoning into the testing and refinement of design solutions. In addition, developing and empirically examining a more integrated theoretical framework for mathematics-focused DT in STEM teacher education could advance understanding of how mathematical reasoning operates across the phases of the design process.

At the curricular and policy levels, the findings highlight the potential value of structures that support iterative, student-centered learning and interdisciplinary collaboration among mathematics, science, and technology educators [2]. Incorporating DT into curriculum frameworks, teacher education standards, and assessment policies may support its more sustained integration into mathematics-focused STEM education [20]. In particular, assessment approaches that recognize the design process, creativity, mathematical reasoning, and evidence-based refinement, as well as the quality of final solutions, may better align assessment practices with the iterative nature of DT. Such changes could help create conditions in which mathematics functions not only as content embedded in design tasks but also as an analytical resource for evaluating and improving design solutions.
